# *Mucor circinelloides* Thrives inside the Phagosome through an Atf-Mediated Germination Pathway

**DOI:** 10.1128/mBio.02765-18

**Published:** 2019-02-05

**Authors:** Carlos Pérez-Arques, María Isabel Navarro-Mendoza, Laura Murcia, Carlos Lax, Pablo Martínez-García, Joseph Heitman, Francisco E. Nicolás, Victoriano Garre

**Affiliations:** a Departamento de Genética y Microbiología, Universidad de Murcia, Murcia, Spain; b Department of Molecular Genetics and Microbiology, Duke University Medical Center, Durham, North Carolina, USA; Vallabhbhai Patel Chest Institute; University of Melbourne; University of British Columbia

**Keywords:** emerging pathogens, host-pathogen interaction, innate immunity, mucormycosis, transcriptomics

## Abstract

Mucormycosis is an emerging fungal infection that is often lethal due to the ineffectiveness of current therapies. Here, we have studied the first stage of this infection—the germination of Mucor circinelloides spores inside phagocytic cells—from an integrated transcriptomic and functional perspective. A relevant fungal gene network is remodeled in response to phagocytosis, being enriched in crucial functions to survive and germinate inside the phagosome, such as nutritional adaptation and response to oxidative stress. Correspondingly, the phagocytic cells induced a specific proinflammatory and apoptotic response to the pathogenic strain. Deletion of fungal genes encoding putative transcription factors (*atf1*, *atf2*, and *gcn4*), extracellular proteins (*chi1* and *pps1*), and an aquaporin (*aqp1*) revealed that these genes perform important roles in survival following phagocytosis, germination inside the phagosome, and virulence in mice. *atf1* and *atf2* play a major role in these pathogenic processes, since their mutants showed the strongest phenotypes and both genes control a complex gene network of secondarily regulated genes, including *chi1* and *aqp1*. These new insights into the initial phase of mucormycosis define genetic regulators and molecular processes that could serve as pharmacological targets.

## Abstract (precis)

Mucorales are a group of ancient saprophytic fungi that cause neglected infectious diseases collectively known as mucormycoses. The molecular processes underlying the establishment and progression of this disease are largely unknown. Our work presents a transcriptomic study to unveil the Mucor circinelloides genetic network triggered in fungal spores in response to phagocytosis by macrophages and the transcriptional response of the host cells. Functional characterization of differentially expressed fungal genes revealed three transcription factors and three extracellular proteins essential for the fungus to survive and germinate inside the phagosome and to cause disease in mice. Two of the transcription factors, highly similar to activating transcription factors (ATFs), coordinate a complex secondary gene response involved in pathogenesis. The significance of our research is in characterizing the initial stages that lead to evasion of the host innate immune response and, in consequence, the dissemination of the infection. This genetic study offers possible targets for novel antifungal drugs against these opportunistic human pathogens.

## INTRODUCTION

Mucormycosis is an angioinvasive fungal infection with mortality rates that can reach up to 90% in cases of disseminated infection (1–3). This infection is caused by diverse species belonging to the order Mucorales, a group of early-diverging fungi (4). The most common species causing mucormycosis belong to the *Rhizopus*, *Mucor*, and *Lichtheimia* genera. These ubiquitous saprophytes can act as opportunistic human pathogens due to the capacity for rapid vascular dissemination with subsequent tissue necrosis (5, 6). Mucormycosis is considered an emerging infection due to a steady increment in incidence among immunocompromised patients, along with the increasing diagnosis of mucormycosis in immunocompetent individuals, especially associated with trauma (7, 8).

A critical aspect of these neglected fungal pathogens is their innate resistance to current antifungal drugs, which partially explains the elevated mortality rates (9, 10). Antifungal therapies with voriconazole and fluconazole have poor outcomes against Mucorales, and other antifungals, such as posaconazole or amphotericin B, are used as salvage therapy and cause serious adverse effects (10, 11). This antifungal resistance in Mucorales may be due to an evolutionarily conserved amino acid substitution in lanosterol 14α-demethylase, the enzyme targeted by short-tailed azole drugs (10). In addition, Mucorales also possess an RNA interference (RNAi)-based resistance mechanism that specifically silences the expression of the gene targeted by the antifungal compound, preventing the formation of a target-antifungal complex that inhibits hyphal growth (9, 12).

Research has focused on finding new targets for antifungal drugs in Mucorales. However, molecular research is challenging because most Mucorales are not amenable to molecular genetic tools. Despite these difficulties, genetic studies in these pathogens have identified dimorphism, iron uptake, spore size, and spore coat proteins as virulence factors in mucormycosis (13–18).

Mucor circinelloides, herein referred to as *Mucor*, offers multiple advantages as a genetic model for pathogenesis among the typically intractable Mucorales. Along with the availability of an efficient genetic transformation procedure, its RNAi mechanism is well characterized and has become a useful tool for functional genetics (19, 20). Thus, an RNAi-based genome-wide screening identified two virulence determinants in mucormycosis: a myosin class V gene involved in intracellular transport and a phospholipase D gene involved in signaling (21). Another advantage of this model is the availability of two wild-type strains with clear, distinct pathogenic potentials in wax moth (16) and mouse (21) models. Indeed, a comparative genomic analysis revealed specific genomic variations between both pathotypes, which could be associated with virulence (22).

Mucoralean asexual spores can reach host tissues via inhalation or open wounds, which are the most frequent routes of colonization leading to mucormycosis. Once inside the host, the spores’ ability to germinate and invade the host defines the establishment of the infection (23). The innate immune response is the first barrier at the site of the infection, where phagocytic cells are recruited to internalize spores and form granulomas in rabbit, mouse, and zebrafish models (24–26). Phagocytosis prevents spore germination in these animal models, and failure of this early immune response leads to disseminated infections (23). Thus, deficiencies in innate immunity are risk factors associated with mucormycosis (2, 27), emphasizing the interaction between Mucorales and their hosts as a crucial stage in the invasive development of mucormycosis.

The molecular and cellular responses of Mucorales to host phagocytic cells are not well defined. Characterization of genes differentially regulated at this stage of the infection by transcriptomic analysis has been postulated as a straightforward strategy to elucidate key molecular aspects of the interaction. The first host-mucoral transcriptomic study was conducted in the fruit fly, in which several host genes related to pathogen recognition and stress response were selectively downregulated after infection (28). Other transcriptomic studies with airway epithelial cells and *Rhizopus* and *Mucor* species revealed that platelet-derived growth factor receptor B signaling participates in the core response to mucoralean infection that damages barrier host cells (29). However, these studies were mainly focused on the host response, and no follow-up analysis of fungal genes was performed.

Here, we conducted an integrated genetic study to detail the response of *Mucor* to host innate immunity. After macrophage phagocytosis, a transcriptomic analysis of spores from two different *Mucor* pathotypes revealed an intricate gene response that could account for the distinctive features of mucormycosis. In particular, a functional enrichment analysis of this response exposed biological and molecular functions required to survive phagocytosis. Deletion mutants of key genes participating in these functions were severely compromised after phagocytosis by macrophages and showed decreased virulence in a murine model. Transcriptomic analyses on mutants in transcription factors involved in virulence revealed a secondary gene network during the initial interaction between macrophages and germinating spores. Taken together, this work contributes to a better understanding of the mechanisms by which members of Mucorales overcome innate immune responses to cause mucormycosis, providing putative targets for new antifungal therapies against this often fatal infection.

## RESULTS

### *Mucor* pathotypes coordinate different transcriptional responses against macrophage phagocytosis.

Previous host-pathogen survival assays have shown significant differences in virulence between two *Mucor* strains. Immunosuppressed mice infected with the R7B strain show high mortality rates, whereas the NRRL3631 strain is completely avirulent (22). The virulence of these strains is related to their spores’ ability to germinate inside host phagocytic cells, since the virulent R7B strain is able to germinate and survive within mouse macrophages while NRRL3631 shows a strong delay in germination (21). To explore the transcriptional differences between the strains in response to host innate immunity, we performed an *in vitro* host-pathogen RNA-sequencing experiment on fungal spores interacting with mouse macrophages. R7B or NRRL3631 spores were cocultured with a cell line of mouse macrophages (J774A.1; ATCC T1B-67) for 5 h, ensuring that all the spores were phagocytized and able to start the germination process inside the macrophages. At this point, the differences in germination inside the phagosome between strains were most evident. Indeed, R7B spores had fully undergone the germination process and produced germ tubes, while NRRL3631 spores were still in a stage of isotropic growth. Noninteraction controls consisted of single cultures of either fungal spores or uninfected macrophages in the same cell medium used in the interaction. These controls were used to exclude differences in gene expression in response to the medium. Total RNA was isolated from both fungus-host interaction and control samples and then sequenced to perform a transcriptomic analysis.

**FIG 1:**
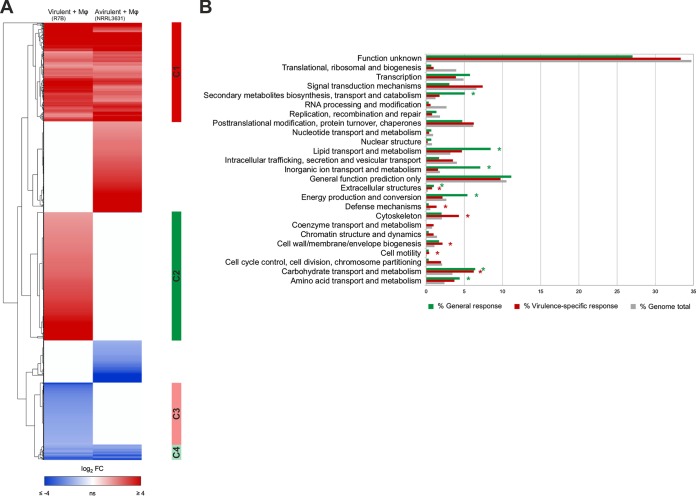
*Mucor* response to mouse macrophages. (A) Heat map showing *Mucor* genes that are differentially expressed (*Q *≤* *0.05) in virulent R7B and avirulent NRRL3631 spores phagocytosed by mouse macrophages (Mφ) (cell line J774A.1) during coculture for 5 h in L15 cell culture medium. Differential expression values with respect to noninteraction cultures (without macrophages) were calculated for each strain and hierarchically clustered according to similarities. Red and blue represent up- and downregulated genes, respectively. Clusters 1 (C1) and 4 (C4) contain genes up- and downregulated, respectively, in both *Mucor* strains during phagocytosis, which was defined as the general response. Clusters 2 (C2) and 3 (C3) contain genes up- and downregulated, respectively, in the virulent *Mucor* strain R7B during phagocytosis, comprising the virulence-specific response. (B) Functional enrichment analysis showing the percentages of genes for each KOG class found in the *Mucor* genome (gray) and in the general (green) and virulence-specific (red) responses. Asterisks (*) indicate a significant enrichment (*P *≤* *0.05, Fisher’s exact test) in the corresponding KOG class with respect to the expected number of genes found in the *Mucor* genome. FC, fold change.

This analysis identified 1,068 genes (>9% of total genes) that were differentially expressed (*Q *≤* *0.05) in the two *Mucor* strains after 5 h of macrophage interaction (Data Set S1 in the supplemental material). Expression values were clustered to discriminate between shared and strain-specific gene sets (Fig. 1A). This classification revealed a general response to the macrophages, comprised of genes that were either up- or downregulated in both the virulent (R7B) and the avirulent (NRRL3631) strain (Fig. 1A, gene clusters C1 and C4, respectively). A functional enrichment analysis of this general response determined that nutrient assimilation and metabolism were strongly induced as a result of the interaction, as well as the production of secondary metabolites and extracellular structures (Fig. 1B, general response), suggesting that nutrient competition in an impoverished environment, such as the phagosome, is critical for the spore to begin germination. These cellular processes, though essential, do not explain the exact mechanism for surviving the host innate immune response. We focused closely on the transcriptional profile of the R7B strain to characterize a virulence-specific response, which contained 464 genes that were differentially expressed in response to the macrophages (Fig. 1A, clusters C2 and C3). Gene clusters involved in the virulence-specific response were enhanced in functions associated with carbohydrate metabolism, cell wall and membrane biogenesis, cytoskeleton, cell motility, extracellular structures, and defense mechanisms (Fig. 1B, data for virulence-specific response). Moreover, we discovered an increase in biological processes involved in regulating cAMP-dependent intracellular signal transduction pathways and the response to oxidative stress (Data Set S1). These functions are in accord with the germination and hyphal development processes observed for the phagocytized spores and could explain their ability to survive macrophage attack.

10.1128/mBio.02765-18.8 DATA SET S1 Differentially expressed genes in *Mucor* strains after 5-h interaction with mouse macrophages. Expression values of virulent *Mucor* strain R7B spores (R7BMφ) interacting with mouse macrophages and noninteraction controls (R7Bc) and of avirulent strain NRRL3631 spores (NRRL3631Mφ and NRRL3631c, respectively, for interaction and noninteraction controls). Download Data Set S1, XLSX file, 3.1 MB. Copyright © 2019 Pérez-Arques et al. 2019 Pérez-Arques et al. This content is distributed under the terms of the Creative Commons Attribution 4.0 International license.

### Virulent *Mucor* strain induces inflammation and apoptotic pathways in mouse macrophages.

**FIG 2:**
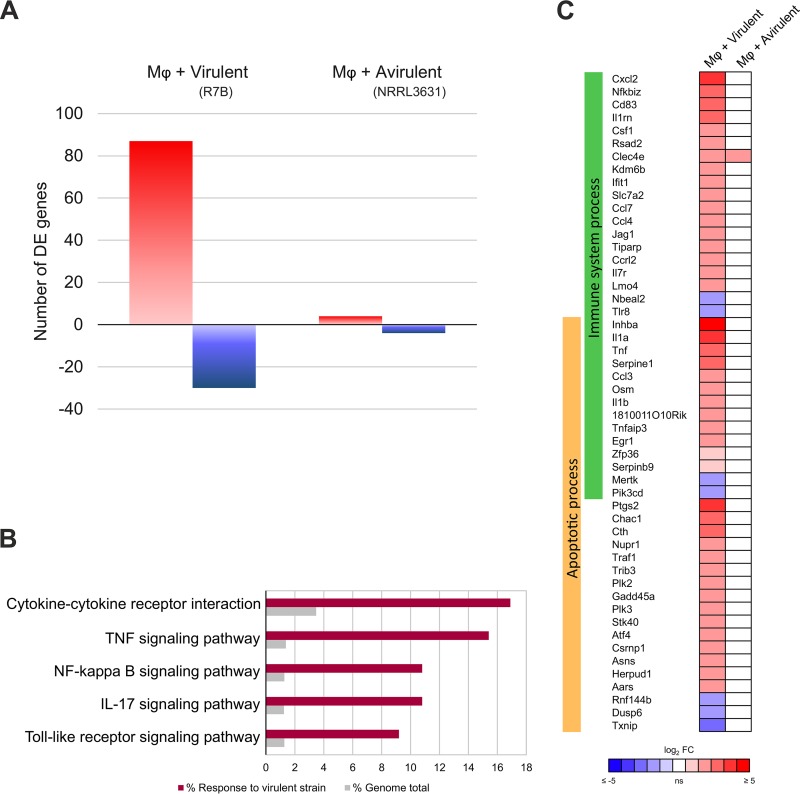
Macrophages respond exclusively to the virulent *Mucor* strain. (A) Differential expression (*Q *≤* *0.05) of upregulated (red) and downregulated (blue) mouse macrophage (Mφ) (cell line J774A.1) genes induced after phagocytosing spores from each depicted *Mucor* strain in L15 cell culture medium for 5 h. Differential expression values were calculated by comparison with expression in noninteraction cultures (without spores). (B) Percentage of genes for each significantly enriched (adjusted *P* value, ≤0.01) KEGG pathway found in the macrophage response to the virulent *Mucor* R7B strain compared to the expected percentage of genes found in the mouse genome. (C) Differential expression values (*Q *≤* *0.05) of selected mouse macrophage (Mφ) (cell line J774A.1) genes induced after phagocytosing spores from each depicted *Mucor* strain for 5 h. The selected genes are involved in two enriched GO Biological Processes (adjusted *P* value, ≤0.05), Immune System Process and Apoptotic Process. FC, fold change.

Gene expression in macrophages responding to each *Mucor* strain was compared to identify a specific reaction to either pathotype that could account for their differences in virulence. A differential expression analysis (*Q* ≤ 0.05) revealed 117 genes that responded to the virulent *Mucor* strain after 5 h of interaction compared to their expression in macrophage cultures without spores. In contrast, reaction to the avirulent *Mucor* strain was largely absent, consisting of 8 differentially expressed genes (Fig. 2A and Data Set S2).

10.1128/mBio.02765-18.9 DATA SET S2 Differentially expressed genes in mouse macrophages after 5-h interaction with *Mucor*. Expression values of mouse macrophages interacting either with virulent *Mucor* strain R7B spores (MφR7B) or avirulent strain NRRL3631 spores (MφNRRL3631) or without spores (Mφ). Genes with a mean FPKM of <1.0 were filtered out. Download Data Set S2, XLSX file, 1.3 MB. Copyright © 2019 Pérez-Arques et al. 2019 Pérez-Arques et al. This content is distributed under the terms of the Creative Commons Attribution 4.0 International license.

A functional analysis of the macrophage response to the virulent strain revealed an enrichment in signaling pathways related to defense against pathogens, particularly inflammation signaling pathways (Fig. 2B). Most of these genes were involved in biological processes related to immunity and apoptosis (Fig. 2C and Data Set S2). This exclusive response to the virulent strain was confirmed by reverse transcription-quantitative PCR (RT-qPCR) of well-known proinflammatory cytokines. Thus, *Il1a* and *Tnf* expression was significantly upregulated in the macrophages (*P* <* *0.005, unpaired *t* test) after 5 h of interaction with the virulent *Mucor* strain (Fig. S1), and their expression was significantly higher than in the cells interacting with the avirulent strain.

10.1128/mBio.02765-18.1 FIG S1 Relative levels of expression of proinflammatory cytokines in response to *Mucor* virulent strain. RT-qPCR-determined expression differences of *Il1a* and *Tnf* mouse macrophage genes during interaction with virulent and avirulent *Mucor* strains. Transcript levels were quantified in cDNA obtained from mouse macrophages (Mφ) (cell line J774A.1) cocultured with either virulent R7B spores or avirulent NRRL3631 spores in L15 cell culture medium for 5 h. Values were normalized using *Actb* as an internal control, and log_2_-fold change differential expression was calculated with respect to expression values in response to the avirulent *Mucor* strain. Error bars correspond to the SD from technical triplicates, and asterisks indicate a significant difference determined by unpaired *t* test (**, *P *≤* *0.005; ***, *P* <* *0.0001). Download FIG S1, PDF file, 0.03 MB. Copyright © 2019 Pérez-Arques et al. 2019 Pérez-Arques et al. This content is distributed under the terms of the Creative Commons Attribution 4.0 International license.

### Genes induced in phagocytosed spores are expressed during *in vivo* infection.

**FIG 3:**
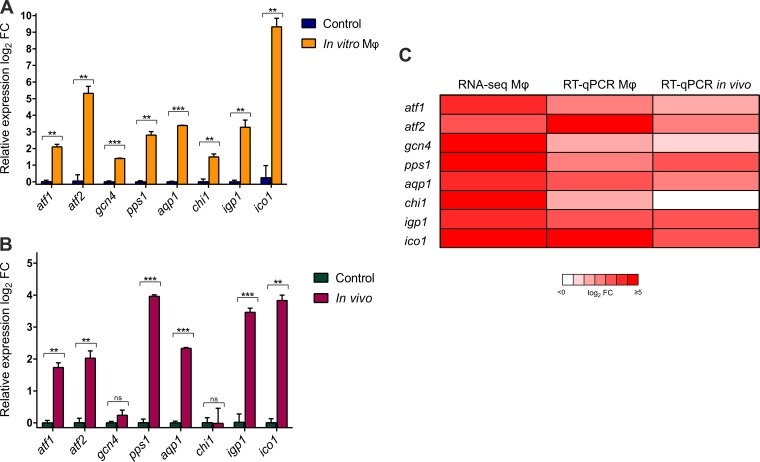
*In vitro* and *in vivo* gene expression analysis of selected *Mucor* genes. (A) RT-qPCR-determined differences in the expression of selected *Mucor* genes during mouse macrophage phagocytosis. Transcript levels were quantified in cDNA obtained from virulent *Mucor* strain R7B spores cocultured with mouse macrophages (Mφ) (cell line J774A.1) in L15 cell culture medium for 5 h and from the same spores cultured in noninteraction controls (without macrophages). Values were normalized using 18S rRNA as an internal control, and the differential expression (log_2_-fold change) was calculated with respect to expression in noninteraction samples. (B) RT-qPCR-determined differences in the expression of the selected genes during interaction with peritoneal macrophages. Transcript levels were quantified in cDNA obtained from virulent *Mucor* strain R7B spores that were injected into the peritoneal cavity of OF-1 mice and recovered after 5 h, as well as from the same spores incubated in L15 cell culture medium as a control. Values were normalized using 18S rRNA as an internal control, and the differential expression (log_2_-fold change) was calculated with respect to the expression in L15 control samples. (C) Comparison of differential expression of selected genes during *in vitro* interaction with mouse macrophages, quantified by both RNA-seq and RT-qPCR, and during *in vivo* interaction with peritoneal macrophages, quantified by RT-qPCR. Error bars correspond to the standard deviations (SD) of the results from technical triplicates, and asterisks indicate a significant difference determined by unpaired *t* test (*, *P *≤* *0.05; **, *P *≤* *0.005; ***, *P < *0.0001).

To validate the transcriptomic response and to further characterize the response of *Mucor* to the host innate immune system, we designed a thorough genetic analysis that combined quantification of both *in vitro* and *in vivo* gene expression with the generation of deletion mutants. The selection criteria focused on genes that were highly upregulated in response to the macrophages (i.e., log_2_-fold change of ≥3.5) and that could regulate the functional categories attributed to the virulence-specific response (Table 1). Thus, we selected the following genes: *atf1* (activating transcription factor 1; Joint Genome Institute *Mucor circinelloides* v2.0 identification number [ID] 190180) and *atf2* (activating transcription factor 2; ID 156289), which encode basic leucine zipper activating transcription factors (ATFs) that could regulate the response to oxidative stress; *gcn4* (general control nonderepressible 4; ID 85517), which encodes a basic leucine zipper transcription factor that could be involved in inducing the robust metabolic response observed in the general response to macrophages; *aqp1* (aquaporin 1; ID 167023), which encodes a putative aquaporin that could be implicated in cell wall and membrane processes; *ico1* (isochorismatase 1; ID 155573), which encodes a putative isochorismatase that could participate in the biosynthesis of siderophores and other extracellular structures and defense mechanisms; and *igp1* (immunoglobulin-like protein; ID 154866), *chi1* (ID 189685), and *pps1* (signal peptide-containing protein; ID 115037), genes encoding proteins of unknown function but with predicted secretion signals that could be Mucorales-specific surface antigens or secreted proteins in direct interaction with macrophages. The expression of these selected genes was analyzed by RT-qPCR, replicating the transcriptome sequencing (RNA-seq) conditions (gene-specific primers are in Table S1). The analysis confirmed the expression pattern of the selected genes, all of which were significantly upregulated (*P* ≤ 0.05, unpaired *t* test) after 5 h of interacting with macrophages, validating the RNA-seq data (Fig. 3A and C).

**TABLE 1:** Candidates for gene deletion

Gene	ID	Secretion prediction	TMHMM^*a*^	Predicted domain (Pfam or InterPro accession no.)	Putative protein homolog (reference) (% identity; alignment score; E value)
*atf1*	190180	No	None	bZIP_1 (PF00170)	Atf1 (45) (30.4; 200; 1.4e−15)
*atf2*	156289	No	None	bZIP_1 (PF00170)	Atf1 (45) (31.1; 180; 5.7e−14)
*gcn4*	85517	No	None	bZIP_1 (PF00170)	GCN4 (62) (50; 100; 1.4e−3)
*pps1*	115037	Yes	None	None predicted	MP88 (40) (29; 190; 7e−17)
*aqp1*	167023	Yes	6	Aquaporin-like (IPR023271)	AQY1 (63) (32; 309; 3.7e−31)
*chi1*	189685	NCS^*b*^	None	None predicted	None
*igp1*	154866	Yes	None	None predicted	None
*ico1*	155573	Yes	None	Isochorismatase (PF00857)	YcaC (64) (61.9; 655; 2.2e−84)

a TMHMM, Transmembrane Helix Prediction based on a hidden Markov Model.

b Putative secretion by noncanonical secretion (NCS) pathway.

10.1128/mBio.02765-18.6 TABLE S1 Primers used in the study. Download Table S1, DOCX file, 0.02 MB. Copyright © 2019 Pérez-Arques et al. 2019 Pérez-Arques et al. This content is distributed under the terms of the Creative Commons Attribution 4.0 International license.

The expression of the *in vitro*-validated genes was further analyzed *in vivo*. Spores of the virulent strain R7B were injected into the peritoneal cavity of mice and recovered after 5 h of interaction with recruited primary phagocytic cells. Total RNA samples were extracted to quantify gene expression by RT-qPCR using the same set of specific primer pairs (Table S1). Genes *atf1*, *atf2*, *gcn4*, *pps1*, *aqp1*, *igp1*, and *ico1* were significantly upregulated (*P* ≤ 0.05, unpaired *t* test) (Fig. 3B). Conversely, *chi1* expression was not affected by the peritoneal phagocytic cells (Fig. 3B). The accurate correlation between *in vitro* expression differences during macrophage phagocytosis and *in vivo* expression for most of the selected genes (Fig. 3C) indicated that the transcriptomic data can be useful in identifying genes that specifically respond to host innate immune cells.

### Deletion of key genes involved in the response to macrophages decreases survival during both phagocytosis and virulence.

Because these genes were highly upregulated in *Mucor* after spores were phagocytosed, we hypothesized that their absence could impact the ability of spores to survive and escape macrophage phagocytosis. To test this hypothesis, single deletion mutants of each of the selected genes were generated by double homologous recombination with a disruption cassette, which replaced their native loci with the *pyrG* gene used as a selectable marker for uridine auxotrophy (Fig. S2). *Mucor* auxotrophic protoplasts were transformed by electroporation with each of the disruption cassettes. Subsequently, transformants that were able to grow on selective medium were selected as putative deletion mutants. Gene deletion was confirmed by PCR (specific primers are in Table S1). Because *Mucor* spores are multinucleated, 10 selective cycles of vegetative growth were conducted to yield homokaryotic deletion mutants, which were confirmed by Southern blot analysis (Fig. S2). To ensure that the phenotypes observed in the mutants were due to deletion of the targeted genes, all further phenotypic assays were conducted with two independently generated mutant strains for each deleted gene (Table S2).

10.1128/mBio.02765-18.2 FIG S2 Disruptions of *atf1*, *atf2*, *gcn4*, *pps1*, *aqp1*, *chi1*, *igp1*, and *ico1* genes. Schematic representation of the wild-type (WT) locus and the mutant (MUT) locus after homologous recombination with the disruption fragment for each gene. Restriction sites for each restriction enzyme used in the Southern blot assays are indicated, showing the expected size of the fragment generated after digestion. The positions of the probes used are indicated for each locus. Southern blot analyses are shown below the schemes. For this, genomic DNA (gDNA) of a WT strain and the gDNA of mutants obtained for each gene were digested with the depicted restriction enzyme. Hybridizations were made with the respective probes, which allowed discrimination between disrupted and WT alleles. The positions and sizes of the GeneRuler DNA ladder mixture (M) (Fermentas) are indicated. Download FIG S2, PDF file, 2.2 MB. Copyright © 2019 Pérez-Arques et al. 2019 Pérez-Arques et al. This content is distributed under the terms of the Creative Commons Attribution 4.0 International license.

10.1128/mBio.02765-18.7 TABLE S2 *M. circinelloides* f. *lusitanicus* strains used in this study. Download Table S2, DOCX file, 0.01 MB. Copyright © 2019 Pérez-Arques et al. 2019 Pérez-Arques et al. This content is distributed under the terms of the Creative Commons Attribution 4.0 International license.

**FIG 4:**
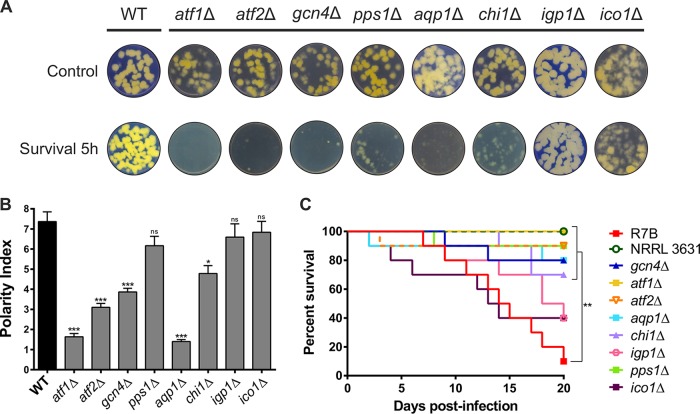
Phenotypic analyses of *Mucor* deletion mutants in selected genes during phagocytosis and mouse infection. (A) Spore fitness of strains with the indicated mutations after 5 h of macrophage phagocytosis, assessed by their ability to develop healthy colonies on MMC medium. Control plates contain spores obtained after growing in cell culture medium for 5 h without macrophages. R7B was used as the wild-type (WT) strain. All MMC plates were incubated for 48 h. (B) Polarity index measures after phagocytosis for all deletion strains generated compared to those of the wild-type control (R7B). Error bars correspond to the standard errors of the means (SEM) from technical replicates (*n* = 50 spores), and statistical significance was analyzed by the unpaired *t* test. (C) Virulence assays with immunosuppressed mice. Each color shows the survival curve for a group of 10 mice infected with 1 × 10^6^ spores from one of the deletion mutant strains. Survival rates were compared to the results for mice infected with a virulent control strain (R7B) and statistically analyzed by a Mantel-Cox test. NRRL3631 was used as an avirulent control strain. Asterisks (*) indicate a significant difference determined by the unpaired *t* test (*, *P *≤* *0.05; **, *P *≤* *0.005; ***, *P* <* *0.0001).

The role of the selected genes in evading the host innate immune response was analyzed using two different approaches. First, the fitness of the mutant strains was assessed after phagocytosis to examine their capacity to endure macrophage phagocytosis. Spores from each deletion mutant strain were cocultured with mouse macrophages for 5 h in mammalian cell culture medium and then plated in minimal medium with Casamino Acids (MMC), pH 3.2, for 48 h to test their growth rates and capacity to develop healthy colonies. Simultaneously, the spores were cultured under the same conditions but without macrophages to ensure that the observed growth defects were exclusively due to the fungus-host cell interaction. Deletion mutants in *atf1* (*atf1*Δ), *atf2* (*atf2*Δ), and *gcn4* (*gcn4*Δ) showed clear defects in growth and development after 5 h of macrophage interaction, while deletion mutants in *pps1* (*pps1*Δ), *aqp1* (*aqp1*Δ), and *chi1* (*chi1*Δ) had defects that were similar but relatively minor (Fig. 4A). On the other hand, deletion mutants in *igp1* (*igp1*Δ) and *ico1* (*ico1*Δ) were able to develop colonies as healthy as those of the wild-type virulent strain.

Second, the germination capacity of the mutant spores was measured inside the phagosome. Polar growth of the deletion mutants was quantified as the ratio between the germ tube length and spore width, considering differences to be significant at a *P* value of ≤0.05 (unpaired *t* test). After 5 h of interaction with macrophages, spores from the virulent strain were fully germinated inside the phagosome, while spores from *atf1*Δ (*P* <* *0.0001), *atf2*Δ (*P* <* *0.0001), *gcn4*Δ (*P* <* *0.0001), *aqp1*Δ (*P* <* *0.0001), and *chi1*Δ (*P = *0.0011) mutants exhibited strong and significant delays in germination (Fig. 4B and Fig. S3). In contrast, *pps1*Δ (*P = *0.1058), *igp1*Δ (*P = *0.3778), and *ico1*Δ (*P = *0.5006) mutants did not show significant differences in polar growth compared to that of the wild-type virulent strain. Together, these germination and developmental defects suggest that the *atf1*, *atf2*, *gcn4*, *aqp1*, *pps1*, and *chi1* genes, which were upregulated during phagocytosis, have a major role in *Mucor* surviving and escaping the phagosome.

10.1128/mBio.02765-18.3 FIG S3 Interaction of macrophages with deletion mutant spores. Micrographs of spores from each deletion mutant after 5 h inside the phagosome of mouse macrophages. Download FIG S3, PDF file, 1.8 MB. Copyright © 2019 Pérez-Arques et al. 2019 Pérez-Arques et al. This content is distributed under the terms of the Creative Commons Attribution 4.0 International license.

*In vitro* defects of the mutant strains could have an impact on virulence because the ability to circumvent the host innate immunity is critical for dissemination. To determine the influence of these genes on infection, we performed virulence assays in a host-pathogen infection model. Immunosuppressed mice were challenged with spores from the mutants, the virulent strain R7B, and the avirulent strain NRRL3631, and survival was monitored for 20 days (Fig. 4C). Differences in survival rates, represented in a Kaplan-Meier curve for each group of mice, were considered statistically significant with a *P* value of ≤0.05 in a Mantel-Cox test. *atf1*Δ (*P* <* *0.0001), *atf2*Δ (*P = *0.0008), *gcn4*Δ (*P = *0.0037), *pps1*Δ (*P = *0.0007), *aqp1*Δ (*P = *0.0021), and *chi1*Δ (*P = *0.0040) mutants were significantly attenuated in virulence compared to the virulent strain R7B; conversely, mutants with *igp1*Δ (*P = *0.1021) and *ico1*Δ (*P = *0.5104) mutations did not show significant differences in virulence. These differences in virulence were replicated in a second virulence assay using independently generated mutants (Fig. S4), supporting the idea that the attenuation in virulence was the result of gene deletion. This correlation between *in vitro* and *in vivo* virulence phenotypes highlights the importance of the fungal response to host innate immunity.

10.1128/mBio.02765-18.4 FIG S4 Virulence assay in mice with independently generated homokaryons. Each color shows the survival curve for a group of 10 immunosuppressed mice infected with 1 × 10^6^ spores from one of the deletion mutant strains. Survival rates were compared to the survival of mice infected with a virulent control strain (R7B) and statistically analyzed by a Mantel-Cox test. NRRL3631 was used as an avirulent control strain. Asterisks indicate a significant difference (*, *P *≤* *0.05; **, *P *≤* *0.005). Download FIG S4, PDF file, 0.06 MB. Copyright © 2019 Pérez-Arques et al. 2019 Pérez-Arques et al. This content is distributed under the terms of the Creative Commons Attribution 4.0 International license.

### *atf1* and *atf2* genes induce pathways related to virulence processes in mucormycosis.

The Atf1 and Atf2 transcription factors appear to be important in the response to host innate immunity, considering that their deletion results in the highest reduction in virulence (Fig. 4C). Knowing which genes are under their control could provide new insights into specific cellular processes involved in virulence. Thus, to increase the scope of the analysis, we analyzed the transcriptomes of *atf1*Δ and *atf2*Δ mutants under two different conditions: cocultured with mouse macrophages for 5 h as previously described and in liquid MMC medium (pH 4.5) for 24 h. Total RNA was extracted, purified, and sequenced as in previous RNA-seq experiments with wild-type strains.

**FIG 5:**
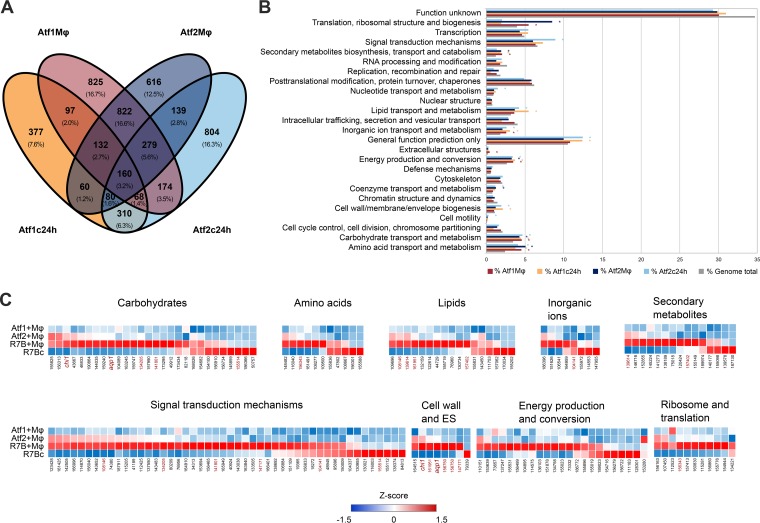
Atf1- and Atf2-regulated genes. (A) Venn diagram showing the relationships between differentially expressed genes (*Q *≤* *0.05) in *Mucor atf1*Δ and *atf2*Δ mutants. Differential expression values were calculated as the ratio between the expression in virulent wild-type strain R7B and in each depicted mutant, either cocultured with mouse macrophages (cell line J774A.1) in L15 cell culture medium for 5 h (Mφ) or grown in liquid MMC, pH 4.5, for 24 h (c24h). Overlapping genes shared similar expression values (up- or downregulation) under the depicted conditions. (B) Percentages of genes regulated by Atf1 (red and orange) and Atf2 (dark and light blue) and of all genes found in the genome (gray) for each KOG class. Asterisks (*) indicate a significant enrichment (*P *≤* *0.05, Fisher’s exact test) in the corresponding KOG class with respect to the expected number of genes found in the *Mucor* genome. (C) Analysis of genes upregulated (*Q *≤* *0.05, fold change of ≥2.0) in the virulent wild-type strain R7B compared to their expression in either the *Mucor atf1*Δ or *atf2*Δ mutant cocultured with mouse macrophages (cell line J774A.1) for each enriched KOG class found in panel B. Z score was calculated as the deviation from the mean FPKM (fragments per kilobase of transcript per million mapped reads) value in standard deviation units for *Mucor atf1*Δ and *atf2*Δ mutants and strain R7B cocultured with mouse macrophages (Atf1+Mφ, Atf2+Mφ, and R7B+Mφ) and for R7B without mouse macrophages (R7Bc). Red gene IDs appear in more than one KOG class.

Under these conditions, 3,384 genes were differentially expressed in the *atf1*Δ mutant compared to their expression in the wild-type virulent strain (R7B). Similarly, 3,644 genes were differentially expressed in the *atf2*Δ mutant (*Q *≤* *0.05) (Data Set S3). These results revealed that *atf1* and *atf2* encode transcription factors that control the expression of a considerable number of target genes. Interestingly, more than 40% of these genes are similarly regulated by both transcription factors (Fig. 5A), suggesting that they function in interacting pathways or are partially redundant.

10.1128/mBio.02765-18.10 DATA SET S3 Differentially expressed genes in *Mucor atf1* and *atf2* mutant strains after 5-h interaction with mouse macrophages. Expression values of *Mucor* R7B wild-type strain and *atf1* and *atf2* mutants interacting with mouse macrophages for 5 h (R7BMφ, Atf1Mφ, and Atf2Mφ, respectively) and grown in solid MMC medium for 24 h (R7Bc24h, Atf1c24h, and Atf2c24h, respectively). Download Data Set S3, XLSX file, 4.0 MB. Copyright © 2019 Pérez-Arques et al. 2019 Pérez-Arques et al. This content is distributed under the terms of the Creative Commons Attribution 4.0 International license.

A functional enrichment analysis of differentially expressed genes was performed to determine the biological processes controlled by *atf1* and *atf2* (Fig. 5B and Data Set S3). As expected by the high proportion of genes regulated by both *atf1* and *atf2*, they appear to share functions. After 5 h inside the phagosome, both transcription factors induced translation and ribosomal processes, carbon and nitrogen metabolism, oxidation-reduction processes, and carotenoid biosynthesis. *atf1* and *atf2* also promoted cell wall and membrane biogenesis and lipid metabolism when the spores were allowed to germinate and grow for 24 h in MMC medium. Regarding nonshared functions, *atf2* controlled specific sets of genes involved in the high-affinity iron uptake mechanism and cytoskeletal changes that could be mediated by cAMP-dependent signaling during macrophage interaction (Data Set S3). High percentages of genes found in these enriched functional categories are upregulated by both transcription factors, being induced in the wild-type virulent strain when challenged by macrophages (Fig. 5C). This analysis revealed that the *chi1* gene is upregulated by Atf1, whereas *aqp1* is upregulated by both Atf1 and Atf2, when *Mucor* spores are internalized by the macrophages.

After phagocytosis, *Mucor* spores must confront a cytotoxic environment within the phagosome that includes acidification, nutrient starvation, oxidation, and antimicrobial proteins. To unveil the role of this ATF pathway in surviving phagocytosis, the germination of the mutants in *atf1* and *atf2* and in their regulated genes was tested at different pHs in yeast nitrogen base (YNB) minimal medium. After a 5-h incubation, *atf1*Δ and *atf2*Δ mutants, as well as *chi1*Δ and *aqp1*Δ mutants, showed significant decreases in their polar growth indices at acidic pH compared to the results for the virulent wild-type strain (Fig. S5A). These differences were not present either when the spores were cultured at neutral pH or in mutants of genes which are not controlled by either ATF (Fig. S5A and B). These results suggest that germination at acidic pH, equivalent to the intraphagosomal environment (30), relies on the ATF pathway.

10.1128/mBio.02765-18.5 FIG S5 Acidic pH affects germination in mutants involved in the ATF pathway. Polarity index measures after 5-h cultures of the depicted mutants in YNB at pH 4.0 (A) and pH 7.0 (B). Error bars correspond to the SEM of the results from technical replicates (*n* = 40 spores), and asterisks indicate a significant difference compared to the results for the virulent wild-type strain as determined by the unpaired *t* test (*, *P *≤* *0.05; **, *P *≤* *0.005; ***, *P* <* *0.0001). Download FIG S5, PDF file, 0.2 MB. Copyright © 2019 Pérez-Arques et al. 2019 Pérez-Arques et al. This content is distributed under the terms of the Creative Commons Attribution 4.0 International license.

## DISCUSSION

In this study, we have identified and functionally characterized the *Mucor* response to host innate immune cells, unveiling key elements involved in mucoralean virulence. The transcriptomic profiles of two *Mucor* strains—one virulent and one avirulent (22)— during mouse macrophage phagocytosis have identified a robust and mutual response between the virulent fungal strain and its host. Prompted by virulent *Mucor* spores, the macrophages induce proinflammatory cytokines and apoptotic processes.

Triggering programmed cell death to evade the immune response is a common mechanism in well-studied fungal pathogens (31–33). Recent studies have described *in vivo* formation of innate granulomas in zebrafish upon *Mucor* infection, formed by phagocytic cells recruited early to the site of infection (34) and apoptosis of infected macrophages (35). The link connecting macrophage apoptosis and the formation of innate granulomas in mucormycosis remains unexplored and represents a promising field to address in future research.

We have thoroughly studied *Mucor* transcriptomic responses to macrophages, revealing for the first time which molecular processes are necessary to survive phagocytosis. Macronutrient metabolic pathways are greatly upregulated in the phagocytosed spores, suggesting that *Mucor* is able to shift toward alternative nutrient sources as a consequence of nutrient scarcity within the phagosome (36). Such changes induced by phagocytosis were previously observed in Candida albicans, which shifts from a glycolytic program to gluconeogenesis, activation of the glyoxylate cycle, and β-oxidation of fatty acids (37). The *gcn4* gene in engulfed *Mucor* spores was upregulated; *gcn4* is a putative homolog of the Saccharomyces cerevisiae gene encoding the essential transcriptional activator of amino acid biosynthesis GCN4 (38). Given that amino acid starvation is a well-studied morphogenetic signal that induces hyphal development in Candida albicans (39), *gcn4* could be orchestrating this metabolic shift that allows *Mucor* spores to fully germinate inside the phagosome and escape phagocytosis. Indeed, *gcn4* deletion causes severe germination and growth defects in the spores after phagocytosis, having a significant impact on the development of mucormycosis in mice.

Surface or secreted proteins could be involved in defense mechanisms and mediate host-pathogen interaction (22). Among genes encoding proteins with a secretion signal, those with no predicted domains were selected under the assumption that they could be specific to Mucorales and therefore participate in the unique properties of mucormycosis. Deletion mutants lacking the *chi1* gene were affected in survival after phagocytosis and germination inside the phagosome, showing a delay in polar growth that could explain their reduced virulence phenotype *in vivo*. Similarly, *pps1*Δ mutants had growth defects after being phagocytosed and a drastic reduction in virulence potential *in vivo.* However, germination in *chi1*Δ mutants was impaired inside the phagosome, whereas *pps1*Δ mutants were not affected in this process. Pps1 is highly conserved among mucormycosis agents (≥70% identity) and is a putative ortholog of the Cryptococcus neoformans immunoreactive mannoprotein MP88, which elicits an immune response in T cells (40). Resembling MP88, Pps1 contains a serine-/threonine-rich region that could serve as a site for extensive O-mannosylation (41), allowing its recognition by macrophage mannose receptors (42). Taken together, these results point to a possible role in mediating cell-to-cell processes involved in the interaction of *Mucor* with macrophages, rather than germination and hyphal growth.

Virulent *Mucor* spores can germinate inside the phagosome, first undergoing an isotropic stage during which spores increase their diameter, followed by rapid germ tube elongation that results in hyphal polar growth (16). During these growth stages inside the phagosome, functions related to cell wall and membrane biogenesis were upregulated in *Mucor* spores, indicating a crucial role in remodeling the cell surface for rapid polar growth inside the phagosome. We generated deletion mutants lacking *aqp1*, a putative aquaporin that could be related to membrane and cell wall modifications during pathogenesis. Several aquaporins are involved during osmotic and oxidative stresses to regulate competitive fitness in fungi (43, 44). Deletion of *Mucor aqp1* resulted in severe defects in germination and polar growth inside the phagosome, suggesting that water or small molecule transport through the cell is essential for *Mucor* spores to start swelling at the isometric stage before hyphal growth.

Two basic leucine zipper transcription factors were highly induced during *in vitro* and *in vivo* macrophage phagocytosis; the genes encoding them were named *atf1* and *atf2* due to their high similarity with Schizosaccharomyces pombe Atf1 (45). ATF/CREB transcription factors are critical for the response to oxidative stress in many fungi, especially filamentous species (46, 47). Both *atf1* and *atf2* are needed to germinate and develop hyphae when the spores are engulfed by macrophages, and the lack of either of them results in a critical decrease in fitness after phagocytosis. The inability of these mutants to escape the host innate immune response leads to a drastic reduction in virulence, highlighting their importance in *Mucor* pathogenesis. As expected, the transcriptomic analyses of these two mutants suggest not only that *atf1* and *atf2* are involved in the response to oxidative stress but also that they participate in macronutrient metabolism. Both transcription factors appear to contribute to the control of these processes, because they regulate a broad number of shared genes in a manner similar to that of *Aspergillus atfA* and *atfB* (48) or S. pombe *atf1* and *pcr1* (49). More importantly, *aqp1* and *chi1* are induced by these transcription factors when *Mucor* spores are phagocytosed, indicating that they are involved in the same signaling pathway which is activated to respond to the macrophages. We hypothesized that this ATF-mediated pathway is induced by the acidic intraphagosomal environment and has a role in germination at low pH, since *aqp1* is regulated by both ATFs and similar mucoralean aquaporins are expressed at acidic pH (50). Indeed, both *atf1* and *atf2* and *chi1* and *aqp1* mutants showed germination defects only at acidic pH, indicating that these genes are involved in the germination process at the phagosomal pH. Our findings support this hypothesis, since mutants in all four genes are also unable to germinate inside the phagosome. This inability to survive the phagosome correlates with a drastic reduction in virulence, suggesting that germinating inside the phagosome at early stages is crucial to evade the host innate defenses and disseminate the infection.

In summary, this study presents a thorough transcriptomic and functional analysis of the interaction of mucoralean spores with phagocytic cells. Mucoralean spores adapt to the host-cell environment by developing an intricate gene response during phagocytosis, which coordinates vital cellular functions involved in germinating within the phagosome and surviving its hostile environment. Indeed, a functional analysis of key genes involved in these cellular functions revealed their role in germinating inside host cells, surviving phagocytosis, and causing infection in mammalian models. Overall, our results provide a better understanding of the molecular mechanisms exploited by these pathogens to evade the innate immune response and invade the host, which could contribute to the development of efficient antifungal therapies.

## MATERIALS AND METHODS

### Fungal strains, cell cultures, and growth conditions.

M. circinelloides f. *lusitanicus* strains are listed in Table S2 in the supplemental material. Strain R7B (51), a leucine auxotroph derived from strain CBS277.49, was used as a wild-type virulent strain, whereas wild-type strain NRRL3631, an environmental isolate, was used as an avirulent strain (22). All mutant strains generated in this work derived from strain MU402 (52), a double auxotroph for uracil and leucine that shows virulence similar to that of its parental strain R7B (53).

*Mucor* cultures were grown at 26°C in yeast nitrogen base (YNB) medium (19), minimal medium with Casamino Acids (MMC) (52), or rich yeast extract-peptone-glucose (YPG) (54) medium, with pH 4.5 for optimal growth and sporulation or pH 3.2 for colonial growth. Media were supplemented with uridine (200 mg/liter) or leucine (20 mg/liter) when required for auxotrophy. For germination assays, YNB liquid medium was adjusted to pHs 4.0 and 7.0 using 1 M NaOH or HCl.

*In vitro* host-pathogen interaction assays were prepared by coculturing a mouse macrophage cell line (J774A.1; ATCC TIB-67) with *Mucor* spores in cell culture medium L15 (Capricorn Scientific GmbH) at 37°C, supplemented with 10% fetal bovine serum (FBS). Amounts of approximately 1.5 × 10^7^ spores of the designated strains were added to the cell cultures, maintaining a proportion of 1.5 spores per macrophage cell. For noninteraction control samples, the same amounts of *Mucor* spores and macrophage cells were cultured separately under the same conditions as the cocultures.

### RNA sequencing and analysis.

*In vitro* host-pathogen interaction assays were cultured for 5 h with macrophages and spores from designated *Mucor* strains, along with their appropriate noninteraction controls. When required as an additional control condition, *Mucor* cultures were grown in liquid MMC (pH 4.5) at 26°C for 24 h. Two replicates of each coculture and control samples were scraped from the plates, and total RNA was extracted using the RNeasy plant minikit (Qiagen, Hilden, Germany) following the supplier’s recommendations. mRNA enrichment by poly(A) purification capture and cDNA libraries were prepared at BaseClear (Leiden, The Netherlands) using TruSeq RNA library preparation kits. The cDNA library was sequenced using the Illumina HiSeq 2500 system.

Quality control of the sequence data sets was conducted with FastQC and Trim Galore! (http://www.bioinformatics.babraham.ac.uk/projects/). Since coculture data sets contained a mixture of fungal and mouse sequences, reads were aligned to each of the respective reference genomes, M. circinelloides version 2.0 and Mus musculus GRCm38, using STAR (55). Differential expression analyses were performed by CuffDiff (56) to calculate significant log_2_-fold changes in gene expression. The M. circinelloides version 2.0 consensus gene annotation or Mus musculus mm10 gene annotation was used accordingly to provide functional information on the gene sets from each species. Functional enrichment of Gene Ontology (GO) terms and KEGG pathways was determined in selected mouse gene sets using g:Profiler (57), while enrichment of Eukaryotic Orthologous Groups (KOG) classes, GO temrs, and KEGG pathways was analyzed on selected *Mucor* gene clusters at FungiDB (58).

### RT-qPCR gene expression analysis.

The expression values of selected genes were measured by RT-qPCR, both *in vitro* and *in vivo*. Total RNA was isolated from *in vitro* host-pathogen interaction replicates in which *Mucor* wild-type strains R7B and NRRL3631 and *atf1*Δ and *atf2*Δ mutants were cultured with mouse macrophages, as described above.

For *in vivo* quantification of gene expression, amounts of 1 × 10^7^ spores of each tested strain were injected intraperitoneally into OF-1 mice. Mice were euthanized 5 h postinjection, and 5 ml of L15 cell culture medium was injected into the peritoneal cavity to recover primary macrophage cells. Peritoneal fluid was collected after a brief abdominal massage and examined under the microscope for cell identification and fungal spore quantification. Total RNA was isolated from pooled samples from three infected mice for each fungal strain.

For RT-qPCR assays, cDNA was synthesized from 1 µg of total RNA using the iScript cDNA synthesis kit (Bio-Rad). Real-time PCR was performed in triplicate with a QuantStudio real-time PCR system (Applied Biosystems) using 2× SYBR green PCR master mix (Applied Biosystems) following the supplier’s recommendations. Primer sequences are shown in Table S1. In order to confirm the absence of nonspecific amplification, a nontemplate control and a melting curve were included. The efficiency of the target gene amplification and the efficiency of the *18S RNA* gene (endogenous control) amplification were approximately equal, so the relative expression of the candidate gene could be obtained using the cycle threshold (ΔΔ*C_T_*) method, normalizing for *18S RNA*.

### Mutant generation by genetic transformation.

Single deletion mutants were obtained by gene replacement through homologous recombination. Disruption fragments contained a 2-kb fragment of the selectable marker *pyrG* flanked by 1-kb upstream and downstream sequences of the targeted gene’s open reading frame. These fragments were PCR amplified separately and then fused by overlapping PCR using specific primers for each deleted gene (Table S1).

MU402 (*pyrG*^−^ *leuA*^−^) protoplasts were transformed by electroporation with approximately 5 µg of a linear disruption fragment following a standardized procedure (59). Transformed protoplasts were grown in MMC selective medium (i.e., without uracil). Genomic DNA (gDNA) was extracted from selected uracil-prototrophic transformants as previously described (18). Deletions were confirmed by PCR amplification with discriminatory primers (Table S1). Mutants underwent 10 cycles of vegetative sporulation and single-colony isolation in selective medium to achieve homokaryosis, which was confirmed by Southern blotting. Specific DNA probes that discriminate between the wild-type and mutant alleles of each gene were obtained by PCR amplification and labeled with [α-^32^P]dCTP using Ready-To-Go labeling beads (GE Healthcare Life Science). After stringent hybridization (60), membranes were developed in a phosphor screen using a Personal Molecular Imager system (Bio-Rad).

### *In vitro* host-pathogen survival assay.

The survival of all mutants was analyzed by conducting *in vitro* host-pathogen assays as described previously. Spores from two independently generated homokaryotic mutants for each gene were added to the macrophage cells (four interaction replicates per deleted gene). After 30 min of incubation, wells were washed with phosphate-buffered saline (PBS) to ensure that every spore remaining was phagocytosed. Then, the interaction was maintained for 5 h. As a noninteraction control, spores were incubated for 5 h in the same cell culture medium but without macrophages. Then, 10 micrographs were taken from each well in which the 5-h-interaction assay was conducted to measure 50 germinating spores using ImageJ software. The polarity index was calculated as the quotient between spore length and width; significant differences were estimated with an unpaired *t* test. Subsequently, macrophages were lysed with 0.1% NP-40 (Sigma-Aldrich) to release *Mucor* spores, which were collected and plated in MMC medium at pH 3.2 (three replicates of 500 spores per independent mutant and condition). The survival of the spores was assessed by their ability to germinate and develop healthy colonies after 48 h of incubation at 26°C.

### Virulence assays.

OF-1 male mice weighing ≥30 g (Charles River, Barcelona, Spain) were used as the host model for virulence assays. Groups of 10 mice were immunosuppressed 2 days prior to infection by intraperitoneal administration of cyclophosphamide (200 mg/kg of body weight) and once every 5 days thereafter. Animals were housed under established conditions with free access to food and autoclaved water. Groups of mice were challenged intravenously with suspensions of 1 × 10^6^ spores collected from each of the mutant or control strains. The survival rate of each group of mice was monitored twice a day, and animals meeting criteria for the endpoint were euthanized by CO_2_ inhalation.

### Ethics statement.

The experiments performed complied with the Guidelines of the European Union Council (Directive 2010/63/EU) and the Spanish RD 53/2013, which is intended to ensure the welfare of animals and the ethics of any procedure related to animal experimentation. Experiments and procedures were supervised and approved by the University of Murcia Animal Welfare and Ethics Committee and the Consejería de Agua, Agricultura, Ganadería y Pesca de la CARM, Spain (authorization number REGA ES300305440012).

### Data availability.

All data discussed in this publication have been deposited in NCBI’s Gene Expression Omnibus (61) and are accessible through GEO Series accession number GSE117385.
