# Supplementary figures and images for: Mucor circinelloides Thrives inside the Phagosome through an Atf-Mediated Germination Pathway
Source: mBio. 2019 Feb 5;10(1):e02765-18. doi: 10.1128/mBio.02765-18 (PMC6428757; doi:10.1128/mBio.02765-18)

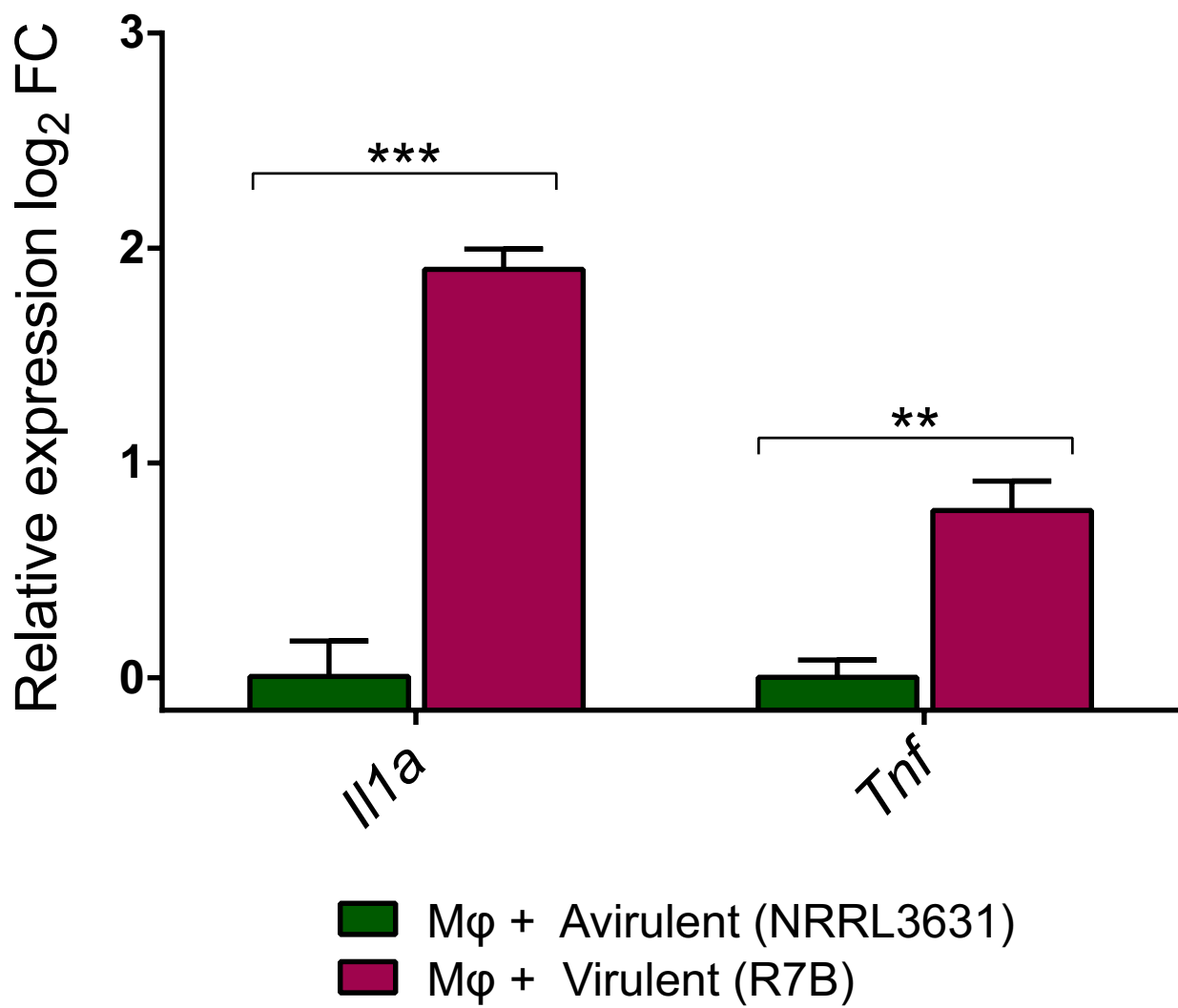

Supplement: FIG S1 [file mBio.02765-18-sf001.pdf]

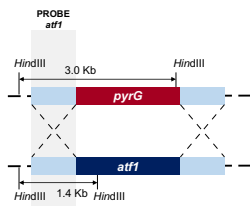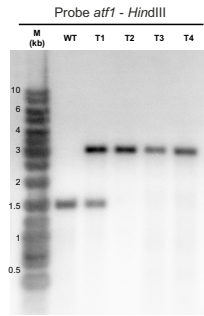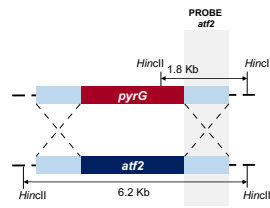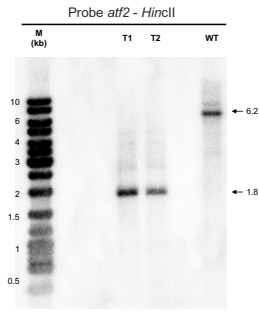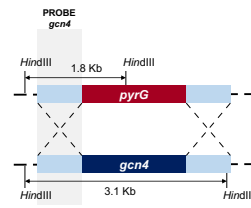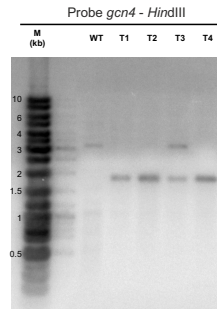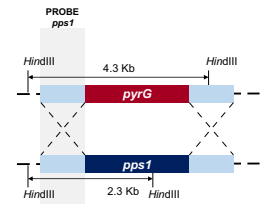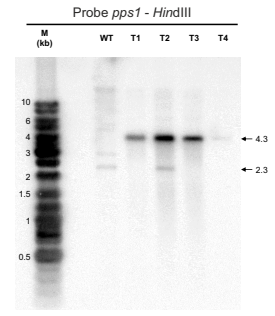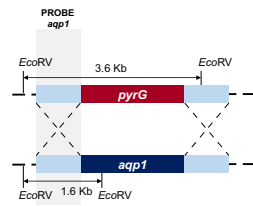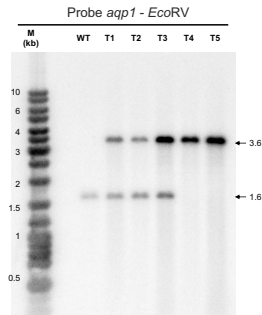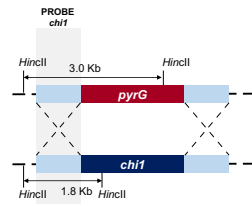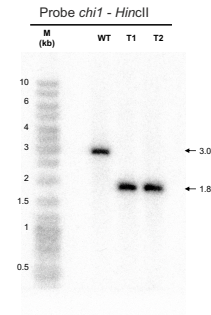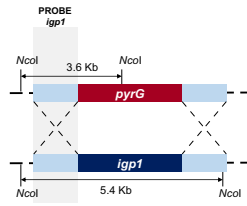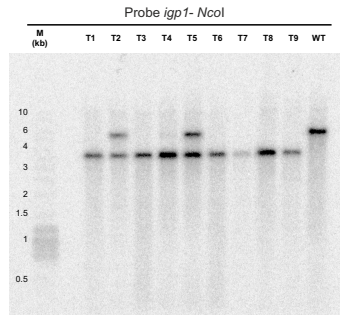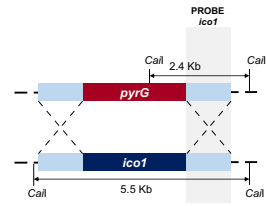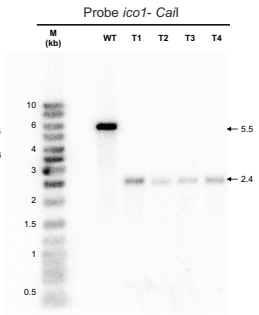

Supplement: FIG S2 [file mBio.02765-18-sf002.pdf]

WT

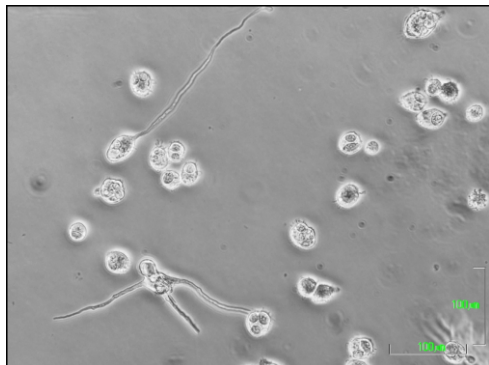

*atf1*Δ

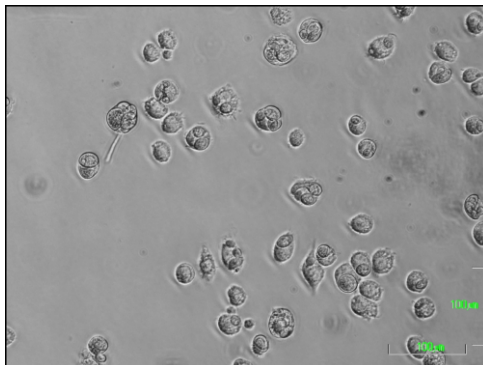

*atf2*Δ

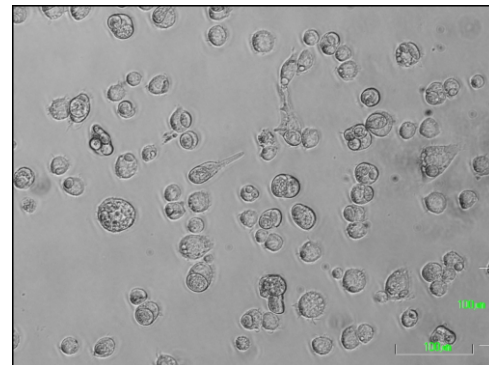

*gcn4*Δ

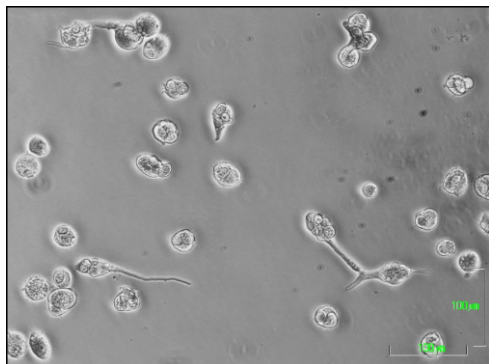

*pps1*Δ

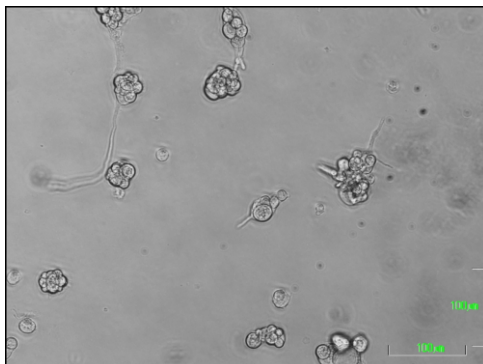

*aqp1*Δ

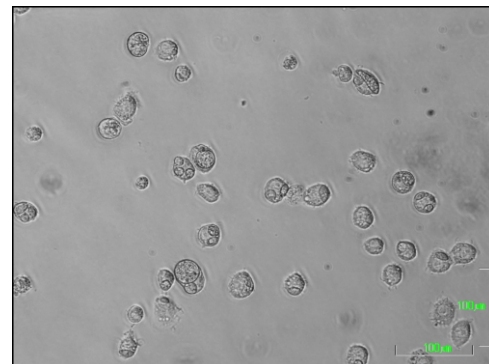

*chi1*Δ

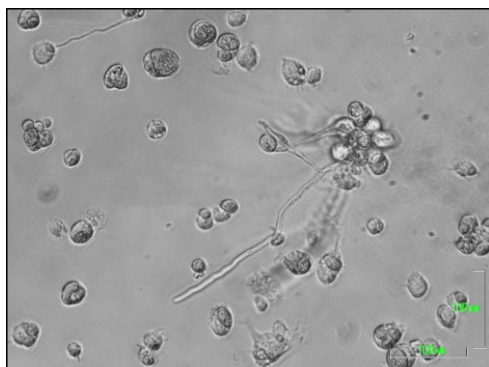

*igp1*Δ

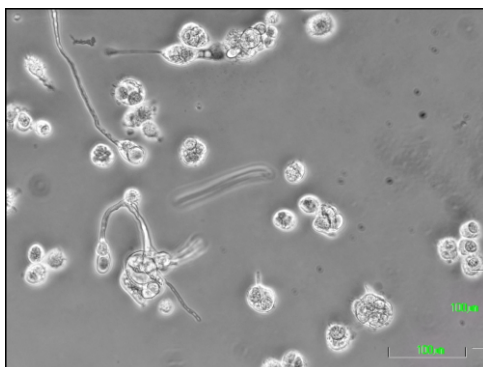

*ico1*Δ

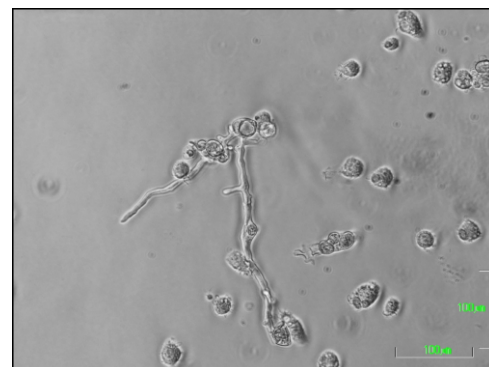

Supplement: FIG S3 [file mBio.02765-18-sf003.pdf]

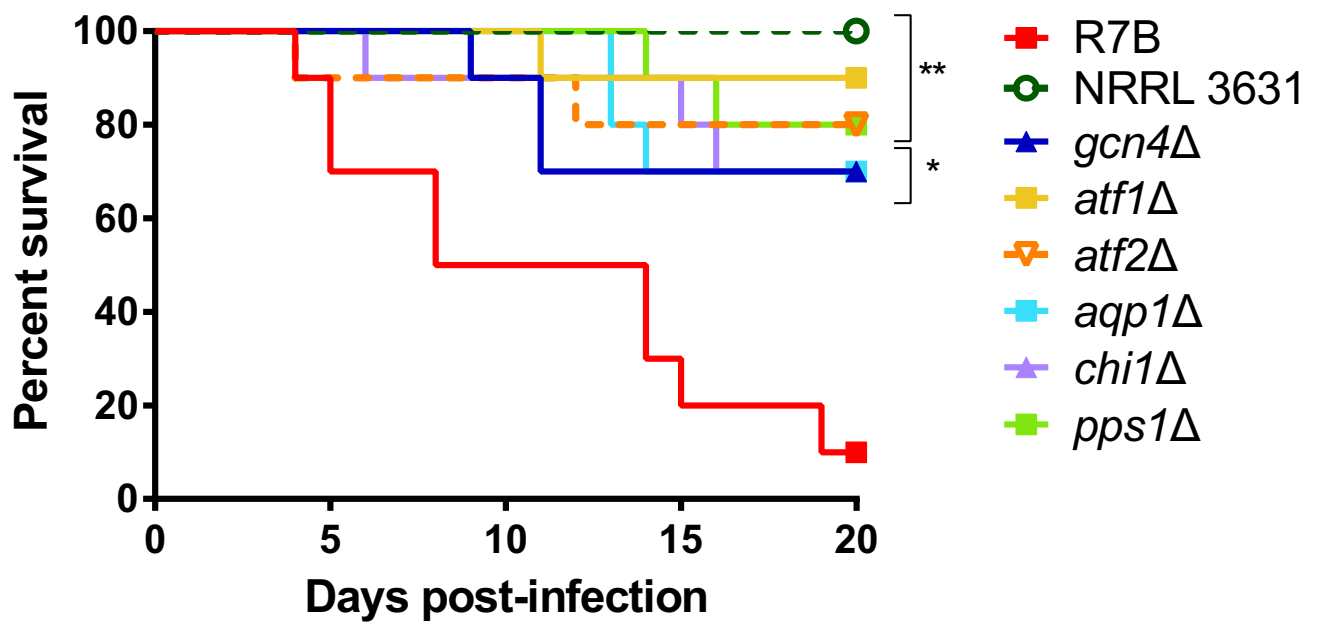

Supplement: FIG S4 [file mBio.02765-18-sf004.pdf]

A

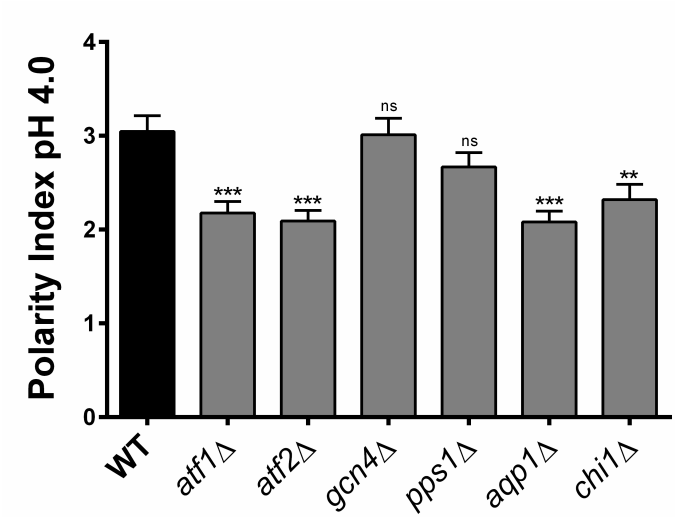

B

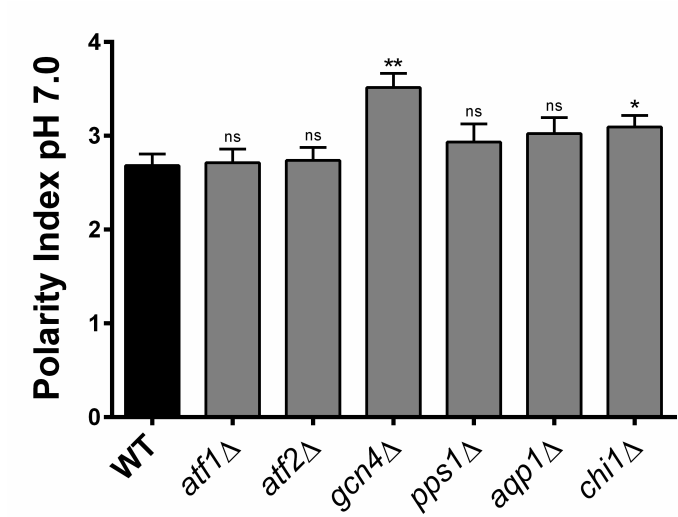

Supplement: FIG S5 [file mBio.02765-18-sf005.pdf]
