# Supplementary material for: Mucor circinelloides Thrives inside the Phagosome through an Atf-Mediated Germination Pathway
Source: mBio. 2019 Feb 5;10(1):e02765-18. doi: 10.1128/mBio.02765-18 (PMC6428757; doi:10.1128/mBio.02765-18)
Supplement: TABLE S1 [file mBio.02765-18-st001.docx]

**S1 Table. Primers used in the study.**

| **NAME** | **SEQUENCE** | **USE** |
| --- | --- | --- |
| atf1UF | ATTGCCAAATTAAGCCGCGATTACC | *atf1* disruption |
| atf1URpyrG | CAAGTACCAATGCTGAGGCATTCTGATGGGCACTGTTTCTCCCTT | *atf1* disruption |
| atf1DFpyrG | CGATAGCATGGCCAGTGTACCACAATGGCCTCTAGAAGAAGCTCA | *atf1* disruption |
| atf1DR | TGCTGCTCTCATGTCTGTTCTCTGA | *atf1* disruption |
| atf1out | GTCAAGAATGTCCTGCCCGTAAGAT | *atf1* mutant comprobation |
| atf1F4 | AATGAAATCCCCTCCCACTC | *atf1* RT-qPCR |
| atf1R4 | TTGTGGAGGTGTATCGACCA | *atf1* RT-qPCR |
| atf2UF | GCGAGGAAAGCGATGAAGATG | *atf2* disruption |
| atf2URpyrG | CAAGTACCAATGCTGAGGCAGCATGGATCACCTCCCATCC | *atf2* disruption |
| atf2DFpyrG | CGATAGCATGGCCAGTGTACACCACAGTTCGGACAAGGC | *atf2* disruption |
| atf2DR | CAGTTTCTGGCGCATCCTTG | *atf2* disruption |
| atf2out | CGAAGCCTGCTTTCATGCTC | *atf2* mutant comprobation |
| atf2F2 | GCAACAAGCGATAAGCAACA | *atf2* RT-qPCR |
| atf2R2 | ATGACGAAGGCACTTGATCC | *atf2* RT-qPCR |
| gcn4UF | TCTTTACTACTGTGGTAGCAGCAGC | *gcn4* disruption |
| gcn4URpyrG | CAAGTACCAATGCTGAGGCAGAAGGAAGAGGGGATGTCTCAAACG | *gcn4* disruption |
| gcn4DFpyrG | CGATAGCATGGCCAGTGTACAAGGAATCTCTTTGCGCTCATCGTC | *gcn4* disruption |
| gcn4DR | GAAGCCATTCCGTATATTGAGATGG | *gcn4* disruption |
| gcn4out | TTGGGCTATGACAATACCATCACAG | *gcn4* mutant comprobation |
| gcn4F4 | GCCGCAAATCAACCACTACT | *gcn4* RT-qPCR |
| gcn4R4 | TTTGAAGGCTCTTGCTCCTC | *gcn4* RT-qPCR |
| pps1UF | ATGACACACTGGAACCTGATTTCAC | *pps1* disruption |
| pps1URpyrG | CAAGTACCAATGCTGAGGCACACTACTGTTGAGTTGCAGCTAGCA | *pps1* disruption |
| pps1DFpyrG | CGATAGCATGGCCAGTGTACGTAACTTGGGCATCTCTACCTATGG | *pps1* disruption |
| pps1DR | ATGATGATTGGAGGCATGGAGTGGA | *pps1* disruption |
| pps1out | GCCTGCAATTTGGCTTTGTAGATGA | *pps1* mutant comprobation |
| pps1F1 | CAGGCTCCTCCTCCTCTTCT | *pps1* RT-qPCR |
| pps1R1 | ATGAGGTGGCATGAAGGAAC | *pps1* RT-qPCR |
| aqp1UF | CGCCACAAGAGAAACGCTGCTAAAC | *aqp1* disruption |
| aqp1URpyrG | CAAGTACCAATGCTGAGGCACCTATTGAGCAGACTCGAGCGATGG | *aqp1* disruption |
| aqp1DFpyrG | CGATAGCATGGCCAGTGTACGCACCTCACTTTGAAGGACAATGGC | *aqp1* disruption |
| aqp1DR | GCCTTTGATCAAGCTAGTAGATAGT | *aqp1* disruption |
| aqp1out | GCAAGCAATCAAATCAAACGAAGCC | *aqp1* mutant comprobation |
| aqp1F2 | GCGTGCTGTGTTTGACAGTT | *aqp1* RT-qPCR |
| aqp1R2 | AAAAGAACGAGCAGGGTTGA | *aqp1* RT-qPCR |
| chi1UF | CGATCTCCACGGTGACAAATCAACT | *chi1* disruption |
| chi1URpyrG | CAAGTACCAATGCTGAGGCACAGTCGATCTGAATTGGGAGTGTGT | *chi1* disruption |
| chi1DFpyrG | CGATAGCATGGCCAGTGTACATTCAATTCTCTCCATGTTCCAGGC | *chi1* disruption |
| chi1DR | CATCGACATTTGACAGCCAGTGCTT | *chi1* disruption |
| chi1out | TACTCTCTCGGCTTTGAGACTGAAG | *chi1* mutant comprobation |
| chi1F1 | TCTTCTCGCCCTGATGAAGT | *chi1* RT-qPCR |
| chi1R1 | TACGACGGAATGCTGACTTG | *chi1* RT-qPCR |
| igp1UF | AGTTGCTGCAGCCTTCCAGACTACGAGTTGTGG | *igp1* disruption |
| igp1URpyrG | CAAGTACCAATGCTGAGGCATTGACAGCGAGTTCTAGCGC | *igp1* disruption |
| igp1DFpyrG | CGATAGCATGGCCAGTGTACAAGGATGGGTTGATGAGGCTTC | *igp1* disruption |
| igp1DR | GCAACCGCGGCCGCCTGAAATCCAACGACGCCATC | *igp1* disruption |
| igp1out | AGCGTTACTGGTTGAGATATCAGG | *igp1* mutant comprobation |
| ipg1F1 | CCCTTTGCTCAATGGTGTTT | *igp1* RT-qPCR |
| ipg1R1 | CGACGGCAGACTCATTGATA | *igp1* RT-qPCR |
| ico1UF | ACGCCTCAAAACACTGATCCT | *ico1* disruption |
| ico1URpyrG | CAAGTACCAATGCTGAGGCATCCACCACCA | *ico1* disruption |
| ico1DFpyrG | CGATAGCATGGCCAGTGTACGCGTTGCGGTTGTGGTAAC | *ico1* disruption |
| ico1DR | GTCAAGCGCCATGAAGAAGC | *ico1* disruption |
| ico1out | TCACTGTATGCCCGAGGTTAC | *ico1* mutant comprobation |
| ico1F1 | TTCGTGATGCTTCTTGGTTG | *ico1* RT-qPCR |
| ico1R1 | GTTGATATCCTTGCGCCAAT | *ico1* RT-qPCR |
| pyrGF | TGCCTCAGCATTGGTACTTG | Selectable marker amplification |
| pyrGR | GTACACTGGCCATGCTATCG | Selectable marker amplification |
| pyrGR2 | ATCCCACCAGAAGGAGTACATGG | PCR mutant comprobation |
| 18sRNAF | CCGACTAGAGATTGGGCTTG | 18s RT-qPCR |
| 18sRNAR | TCTGGACCTGGTGAGTTTCC | 18s RT-qPCR |
| Actb_F1 | CTCTTTTCCAGCCTTCCTTCTT | *Actb* RT-qPCR |
| Actb_R1 | GCTAGGAGCCAGAGCAGTAATCT | *Actb* RT-qPCR |
| Il1a_F1 | TCTGAGAACCTCTGAAACGTCA | *Il1a* RT-qPCR |
| Il1a_R1 | GTATCTCAAATCACTCTGGTAGGTG | *Il1a* RT-qPCR |
| Tnf_F1 | GGACAAACACTATCTCAGCACCA | *Tnf* RT-qPCR |
| Tnf_R1 | AGGTCGGTCTCACTACCTGTGAT | *Tnf* RT-qPCR |
