# Supplementary material for: Mucor circinelloides Thrives inside the Phagosome through an Atf-Mediated Germination Pathway
Source: mBio. 2019 Feb 5;10(1):e02765-18. doi: 10.1128/mBio.02765-18 (PMC6428757; doi:10.1128/mBio.02765-18)
Supplement: TABLE S2 [file mBio.02765-18-st002.docx]

**S2 Table. *M. circinelloides* f. *lusitanicus* strains used in this study**

| Strain | Genotype | # Mutants | # Homokaryons | Homokaryons used for phenotypic analyses | Phenotype |
| --- | --- | --- | --- | --- | --- |
| NRRL3631 | Wildtype | - | - | - | Wildtype, avirulent |
| CBS277.49 | Wildtype | - | - | - | Wildtype, virulent |
| R7B | *leuA*^-^ | - | - | - | Leu-, virulent |
| MU402 | *leuA*^-^, *pyrG^-^* | - | - | - | Leu-, Ura-, virulent |
| *atf1*Δ | *leuA*^-^, *atf1*::*pyrG* | 4 | 3 | MU818^a^ and MU819^b^ | Discussed in this work |
| *atf2*Δ | *leuA*^-^, *atf2*::*pyrG* | 2 | 2 | MU823^a^ and MU824^b^ | Discussed in this work |
| *gcn4*Δ | *leuA*^-^, *gcn4*::*pyrG* | 4 | 3 | MU815^a^ and MU816^b^ | Discussed in this work |
| *pps1*Δ | *leuA*^-^, *pps1*::*pyrG* | 4 | 3 | MU834^a^ and MU835^b^ | Discussed in this work |
| *aqp1*Δ | *leuA*^-^, *aqp1*::*pyrG* | 5 | 2 | MU825^a^ and MU826^b^ | Discussed in this work |
| *chi1*Δ | *leuA*^-^, *chi1*::*pyrG* | 2 | 2 | MU821^a^ and MU822^b^ | Discussed in this work |
| *igp1*Δ | *leuA*^-^, *igp1*::*pyrG* | 9 | 6 | MU830^a^ and MU832^b^ | Discussed in this work |
| *ico1*Δ | *leuA*^-^, *ico1*::*pyrG* | 4 | 4 | MU836^a^ and MU827^b^ | Discussed in this work |

^a^Independently generated homokaryons used in Figures 4 and S3

^b^Independently generated homokaryons used in Figure S4
